# COVID-19 and Asian Americans: Reinforcing the Role of Community-Based Organizations in Providing Culturally and Linguistically Centered Care

**DOI:** 10.1089/heq.2021.0124

**Published:** 2022-03-31

**Authors:** Jennifer A. Wong, Stella S. Yi, Simona C. Kwon, Nadia S. Islam, Chau Trinh-Shevrin, Lan N. Đoàn

**Affiliations:** Department of Population Health, Section for Health Equity, NYU Grossman School of Medicine, New York, New York, USA.

**Keywords:** community-based participatory research, community health workers, health equity, Asian Americans, immigrants

## Abstract

**Introduction:**

Community-based organizations (CBOs) have provided critical resources during the pandemic, particularly for marginalized communities, and are trusted liaisons who connect socially and linguistically isolated community members, such as the highly diverse Asian American population, to care during public health emergencies. Stereotypes such as the model minority myth have permeated public perception of Asian Americans' health status and health care access needs, fueling widespread belief that Asian Americans do not experience health disparities, and mask the high rates of coronavirus disease 2019 (COVID-19) infection, hospitalization, and mortality among Asian Americans. The unequal burden of COVID-19 on Asian American communities has largely remained absent from the public health and national discourse, with exceptions such as community voices that have directed news media coverage and leading roles of CBOs in offering culturally adapted, in-language programming on COVID-19 infection prevention and control.

**Methods:**

CBOs and their staff are well-equipped with the cultural acuity, language capacity, and familiarity with local norms to improve structural gaps affecting health outcomes and support health care delivery.

**Results:**

We discuss the roles and responsibilities of CBOs in strengthening the health care workforce and expanding community-clinic linkages and provide two case studies illustrating the efforts of two community organizations serving Asian American and immigrant communities, who have been disproportionally affected by the COVID-19 pandemic.

**Discussion:**

CBOs are essential to supporting health service coordination and care delivery for structurally vulnerable populations, and are vital to sustaining the coordinated, multilevel public health response to improving community health.

**Conclusion:**

Bolstering the current infrastructure to support CBOs is necessary to facilitating immediate responses to serve community needs.

## Introduction

The coronavirus disease 2019 (COVID-19) pandemic has drastically changed the delivery of inpatient and outpatient care in health care organizations. More than 4.6 million people worldwide have died due to COVID-19, with 14% of deaths originating in the United States,^1^ and the health care gap in COVID-19 infection and prevention resources and necessary medical care continues to widen for historically vulnerable populations, who bear the disproportionate brunt of COVID-19 infections, hospitalization rates, and deaths.^2^

One solution to enhancing direct health care support during public health emergencies is through engagement with community-based organizations (CBOs), who are well-recognized for curbing the spread of COVID-19 infection.^3^ Specifically, CBOs are particularly suited to leveraging their deep understanding of the local community to help bring people to point of care, such as in offering programs and services to support health insurance enrollment, provide patient navigation of health care systems, improve language accessibility in clinical settings, and offer general psychosocial support throughout these processes.

CBOs have the capacity to prepare and coordinate direct support and culturally resonant coaching that have been critical to bridge communities to COVID-19 testing and vaccination sites and to address nonclinical concerns.^4,5^ As gatekeeping organizations to the communities they serve, they are a critical link for building community trust in existing health systems and increasing the uptake and acceptability of COVID-19 vaccines as vaccine deployment continues.

## COVID-19-Related Health Disparities Among Asian Americans

The model minority stereotype was invented by the white conservative majority in the 1960s and perpetuates a narrative through which Asian Americans are perceived to be hardworking, and academically and financially successful.^6^ The model minority myth has permeated the public perception of Asian Americans' health status and health care access needs, fueling widespread belief that Asian Americans do not experience health disparities. This perception is evident in patient and clinician biases about Asian American health status, leading, for example, to underscreening for cancers^7^ or other chronic diseases in Asian Americans, and misperception of mental health status^8^ or overlooking of mental health symptoms^9^ in Asian American subgroups.

Asian American populations are also underrepresented in federally funded clinical trials,^10^ which, when compounded with a lack of sustained investment in race/ethnicity data disaggregation efforts in research methodology, data collection, and data reporting, only further elevates the experiences and identities of some groups while masking the real-world experiences of others.^11^

The model minority myth enables inaccurate assumptions that Asian Americans are monolithic, and that health disparity research in these populations is less needed,^12^ and by extension that culturally or linguistically tailored COVID-19 messaging and outreach efforts are less critical for these populations, thereby working to systematically mask COVID-19-related health disparities and the pandemic's impact on Asian American communities.

Moreover, the racial/ethnic data needed to counter the misrepresentation of Asian American health outcomes, particularly those experienced during the pandemic, are infrequently collected or reported, due to insufficient sample sizes or lack of disaggregated race/ethnicity data. Empirical data have demonstrated high COVID-19 cumulative incidence and case fatality rates, and a high number of excess deaths nationally, among Asian Americans,^13^ yet the attention placed on Asian Americans and COVID-19 has been minimal in proportion to the burden.

As of January 20, 2022, the Centers for Disease Control and Prevention (CDC) reported that Asian Americans accounted for ∼3.6% of COVID-19 cases and 3.4% of COVID-19 deaths.^14^ However, demographic trends for COVID-19-related data only represent the percentage of COVID-19 data where race/ethnicity are available. Evidence also suggests that low testing rates, greater disease severity, socioeconomic factors, and racial discrimination may contribute to COVID-19 disparities among Asian Americans.^13^

The available data on the impact of COVID-19 among racial and ethnic minority communities in COVID-19 hotspot counties are also undercounted due to poor surveillance data, race/ethnicity misclassification, and limited data on ethnic subgroups.^15^ Estimates indicated a high burden of COVID-19 deaths among Asian Americans, with almost 14,000 excess deaths; Asian Americans have the second-highest increase in deaths following Hispanic Americans, using data from July of 2020.^16^ Similarly, high rates of COVID-19 infection, hospitalization, and mortality among South Asian and Chinese American adults have been reported,^17^ highlighting the heterogeneous health outcomes within the “Asian American” umbrella category.

In contrast to the model minority myth, social disadvantages persist across Asian American communities, many of whom experience high rates of limited English proficiency (LEP) and foreign-born status, which are major barriers to health and preventive care access.^18^ Compared with English-proficient individuals, individuals with LEP are more likely to lack a primary place of care, report unmet medical care needs, and experience communication challenges when in health care settings; however, most low-literacy strategies have focused on English- and Spanish-speaking populations.^19^

Decades of anecdotal evidence and empirical research underscore the importance of health literacy, yet COVID-19 health information continues to be presented in ways that are difficult for the public to understand and act upon.^20^ Culturally tailored and in-language community health worker (CHW)-led health promotion interventions have been found to successfully increase the uptake and awareness of the benefits of preventative screenings among racialized populations, including Asian Americans, emphasizing the significance of key messengers.^21^

## Role of CBOs in Bolstering Asian American Community Health

CBOs and their staff are well-equipped with the cultural acuity, language capacity, and familiarity with local norms to improve structural gaps affecting health outcomes and support health care delivery. For example, community health centers and social service organizations often played a leading role in offering culturally adapted, in-language programming on COVID-19 infection prevention and control, to fill the lacking response from federal organizations and government assistance. Community voices have also directed news media coverage to the unequal burden COVID-19 has had in Asian American, Native Hawaiian, and Pacific Islander (NH/PI) communities, which remains absent from the public health and national discourse.

CBOs serve as community advocates to engage historically disenfranchised communities and can improve information-sharing and navigation of the health care systems to prevent and control the spread of COVID-19 infection and misinformation.

### National Advisory Committee model to guide research priorities

At the NYU Center for the Study of Asian American Health (CSAAH), our research and approaches are guided by an integrative population health equity framework.^22^ CSAAH's COVID-19-related efforts have been heavily informed by our National Advisory Committee (NAC) on Research, to ensure that community voices are interwoven in CSAAH's research initiatives, resource development and dissemination strategies, and capacity-building resources.

The NAC is co-led by CSAAH and the Asian and Pacific Islander American Health Forum (APIAHF), an established national health advocacy and policy-oriented agency. The NAC includes leaders representing community-based, social service organizations, professional organizations, and local advocacy agencies supporting Asian American and NH/PI communities.

Several NAC organizations have received designation as federally qualified health centers (FQHC), allowing them the ability to provide primary and preventative health services in-house at community health centers, as part of their social service offerings. For example, the Center for Pan Asian Community Services (CPACS) is an NAC member organization based in Atlanta, GA, that provides immigrant and refugee families culturally and linguistically concordant social and human services. In 2015, CPACS established the COSMO Health Center, an FQHC affiliated with CPACS, that provides comprehensive health care services to low-income, uninsured, and limited English-proficient patient populations, in response to increasing community demand and growing organizational capacity.

NAC member organizations provide a wealth of social services, health education, and community outreach that underscore deep ties and familiarity with local communities and the longstanding trust these organizations often carry with the communities they serve. Table 1 describes NAC organizations' community COVID-19 response efforts, including culturally relevant, no-cost food provision, free COVID-19 testing at local supermarkets and churches, and neighborhood pop-up vaccination sites. Throughout the COVID-19 pandemic, NAC organizations have also shared critical real-time insights and on-the-ground knowledge about how their services and programming have pivoted, such as the rapid transition to virtual health services.

**Table 1.** Examples of National Advisory Committee (NAC) Programming During the Pandemic

NAC member organizations	Populations served	Clinical and nonclinical services and resources provided
Apicha Community Health Center	Asian Americans, Pacific Islanders, and underserved or “otherized” communities in NYC	Apicha's FQHC quickly transitioned to providing telehealth/virtual appointments as of March 2020 to ensure continuity of care and also able to provide walk-in PCR testing to its patient population. Regularly hosts free vaccination pop-up events at neighborhood sites across NYC boroughs, in partnership with local businesses and CBOs (food bazaar, places of worship, community centers, etc.). Provides plain language explanation to address COVID-19 virus and vaccine myths and misinformation to different subsets of community via social media channels. On-staff clinical providers and CHWs/patient navigators supporting community outreach to share COVID-19 infection prevention information, encourage testing and vaccination, and offer PPE distribution. Efforts continue to focus on health information sharing to improve and encourage community care-seeking/testing, address vaccine hesitancy, encourage social distancing and protective measures.
Asian Pacific Community in Action (APCA)	Asian Americans, NH/PI, and emerging communities in Phoenix, AZ	Shift to virtual or telephone assistance to support clients enrolling in Medicaid/ACA marketplace health insurance and unemployment benefits. Collaborate with local organizations to train and deploy Community Health Workers in order to (a) address vaccine misinformation in a linguistically and culturally relevant approach, both in-person and virtually, and (b) to overcome language and cultural barriers and assist community residents in connecting to vaccination and testing services across the Phoenix metropolitan area. Hosted Trauma Dialogue Facebook Live series to advocate for oral health and reproductive justice.
Center for Pan Asian Community Services (CPACS)	Immigrants, refugees, and underprivileged communities in Atlanta, GA	Provides free, universal, drive-through COVID-19 and HIV testing for community, testing 600 community members annually. Hosting health fairs to provide HIV testing, vaccines, health screenings, food distribution, and PPE to community members. On-staff clinical providers and CHWs/patient navigators offer in-language COVID-19 infection prevention information, testing materials, and continued support services.
Chinese American Medical Society (CAMS)	Chinese medical and clinical professionals in greater NYC metropolitan area	Provides in-language, web-based community COVID-19 informational webinars with live question and answer periods, and in-language television and radio programs related to COVID-19. Conducts a variety of phone and virtual town hall lectures and webinars on COVID-19 vaccine and on COVID-19 and cardiovascular disease for frontline staff, including 500+ home health aides and 70+ members of the Visiting Nurse Service of NY. Hosts community distribution of free PPE and partnered with local partners and NYC HHS to offer mobile COVID testing in the community.
Council Of Peoples Organization (COPO)	South Asians and Muslim immigrants in NYC	Hosts and operates halal food pantry catering to LEP, low-income residents in Brooklyn, NY. Provides health insurance, immigration, SNAP benefits enrollment, early childhood education, patient navigation, and other social services. Active member of NIH's NY CEAL initiative, working to increase testing and vaccination rates across the NYC South Asian community.
Asian American Health Coalition (AAHC) of the Greater Houston Area, dba HOPE Clinic	Asian Americans, Pacific Islanders, and LEP community in greater Houston, TX	Trained FQHC clinical staff began offering COVID-19 immunizations in December 2020 across four clinic sites in Houston. Provided drive-through COVID-19 PCR testing since May 2020, as well as on-site COVID-19 screening with a specific antibody testing unit. In 2020, HOPE Clinic served 23,355 patients across 155,119 visits, with ∼15% of patient visits conducted virtually or by phone. Staff includes trained providers, clinical staff, and CHW/patient navigators providing primary, pediatric, obstetrics and gynecology, eye, behavioral health, and dental health care services to more than 20,000 unique patients in over 30 languages. Offers ongoing series of virtual health cooking demonstrations for older adults and mental health discussions tailored for adolescents.
India Home^44^	South Asian older adults in NYC	Offering in-language, culturally adapted virtual COVID-19 awareness lectures, and virtual exercise and art classes to facilitate social connectedness and reduce social isolation. Local staff provide culturally competent and nutritious meal delivery (3 times/week) to over 100 older adults and more than 1000 grocery deliveries to more than 500 seniors to address food insecurity. Provides vital COVID-19 resources, including masks, COVID-19 infection prevention information, and contact tracing programming in high areas of need in Queens, NY, and across NYC. Active member of NIH's NY CEAL initiative, working to increase testing and vaccination rates across the NYC South Asian community.
Kalusugan Coalition	Filipino Americans in NY and NJ	Hosts regular #FilAmHealthCOVID19 webinar series covering COVID-19 health education and wellness forum for Filipino American community (18 archived sessions, more scheduled). Leveraging social media channels to give voice to the contributions and experiences of Filipino RNs working on the frontline, and curating and disseminating in-language (Tagalog) COVID -19 health education materials on KC website. Partnering with the Philippine Nurses Association of American (NY and national chapters), professional agencies to coordinate PPE distribution, infection prevention and control support, and wellness provision to Filipino frontline workers across the United States. Providing strategic and logistical support to Philippine Consulate General in NY to support PPE and vaccine supply distribution to frontline workers, and partnering with local community organizations to address xenophobia and racism against Asian Americans.
Papa Ola Lōkahi	NH/PIs in Hawaii	POL staff actively colead the collaborative NH and PI Hawai'i COVID-19 Response, Recovery and Resilience Team (NHPI 3R) since May 2020, delivering accurate, up-to-date biweekly COVID-19 testing and vaccination data reports on NH/PI communities.^45^ Developed translated public service announcements (PSA) videos and media tailored for NH/PI communities encouraging social distancing, COVID-19 infection prevention strategies, and listing local community testing and vaccination events and sites. Produces biweekly listing of island-specific support services, offers community education materials and in-language guidance resources, disseminated widely in coordination with HI State Health Department and community and research partners. Lead community organization coordinating across Hawai'i County health departments to coordinate testing, information-sharing, and resource delivery through the NHPI 3R effort in alignment with the National NHPI Response Team. Contributing member of a coalition of community-serving partner organizations supporting CDC's Project Firstline, which seeks to promote COVID-19 infection prevention and control across Asian Americans and NH/PI health care workforce members and CDC's Forging Asian, Native Hawaiian, and Pacific Islander Community Partnerships for Rapid Response to COVID-19 Initiative, which aims to address COVID-19 misinformation in Asian American and NH/PI communities in the metropolitan areas of Seattle, WA and New York City, NY, Northern California, and all islands in Hawai'i. Actively informs the development of culturally and linguistically appropriate communication materials and dissemination as a planning member of the National Community Coalition Board (N-CCB) and National Advisory Board (NAB) to Morehouse School of Medicine (MSM)'s National COVID-19 Resiliency Network, a joint initiative funded by the U.S. Department of Health & Human Services Office of Minority Health to collaborate with community-based organizations across the nation to deliver education, information and resources to help fight the pandemic.
Korean Community Services of Metropolitan New York, Inc. (KCS)	Korean American immigrants in NY and NJ	Provides free COVID-19 vaccine and booster doses to all New Yorkers as one of only a few city-designated community-based vaccine sites, through a partnership with NY Health+Hospitals. CBO staff providing culturally competent, weekly meal delivery services and ongoing health, wellness, and community social services and interpretation support to low-income, LEP, immigrant clients, and the broader community. CBO staff providing live, virtual events or short video tutorials to encourage vaccination and address community concerns in-language (such as how to use at-home test kits), reaching over 32,000 social media viewers in November 2021. Active member of NIH's NY CEAL initiative, working to increase testing and vaccination rates in the community, partnering with faith-based organizations to address misinformation and fear related to vaccines in Korean community. In 2020, developed and disseminated translated COVID-19 educational videos with the KAMPANY and CSAAH on: (1) general COVID-19 information; (2) COVID-19 and HBV; (3) COVID-19 and diabetes. Clinical and lay health worker staff providing free COVID-19 PCR testing, vaccination, and antibody screening events at KCS, successfully preregistering and testing more than 1000 individuals across four events in May 2020.
National Tongan American Society (NTAS)	Tongans and Pacific Islanders in Salt Lake City and UT state	Member of PI-CoPCE Islander's National Pacific Islander COVID-19 Response Team Planning Group, launching Strategic Action Plan to support PIs across the United States through primary care services and collaboration with CHCs and CBOs. Through PI-CoPCE, helps disseminate Koviki Talk Podcast for PI communities, families, and allies on COVID-19 and cultural wisdom. Partnered with several UT-local organizations to accelerate the distribution of the COVID-19 vaccines and booster doses to PI community, including coordinating COVID-19 PCR testing, vaccination drives, events, and sites, identifying strategic gathering points in the community, such as at churches and schools, to provide easy community access, including efforts in partnership with the Utah Pacific Islander Coalition, and other partners, to help stop the spread of COVID-19. Collaborated with the Utah Food Bank to distribute free boxes of food to families at clinic locations to address food scarcity in the community, in response to the devastating impact COVID-19 has had on PI families, many of whom are employed as essential frontline workers. NTAS staff and CHWs continue to offer free translation and interpretation assistance, health care and benefit enrollment support and citizenship application help, food security assistance, and social services.^46^ Providing important messaging and ongoing education on issues relating to impact of COVID-19 on traditional cultural practices, such as faikava (kava drinking gatherings), putu (funerals), and lotu (religious gatherings).
Orange County Asian Pacific Islander Community Alliance (OCAPICA)	Asian Americans, NH/PIs, and a broader underserved community in Orange County, CA	Regularly serves 40,000 individuals in 26 languages, with language capacity to support Asian American and NH/PI, Middle Eastern (Farsi, Arabic, Pashtu), and Spanish speakers. Driver of Asian American and NH/PI community outreach and education on free COVID-19 testing (∼300–1000 people/day, 3 days/week) at local community churches, temples, ethnic markets, and community clinics, focused on un/underinsured and essential workers. Also leads contact tracing in partnership with County public health system, local FQHCs, Medicaid Managed Care program. CHWs and CBO staff providing mental health workshops to support OC student and families through virtual education sessions and mental health telehealth services, supporting ∼2000–4000 each month. Partnered with over 11 Orange County school districts to provide mental health support and in-language COVID-19 information for families. Leads coalition of 27 Asian American- and NH/PI-serving organizations to inform local and county clinics' COVID-19-related outreach and infection prevention and control education. Collaborating to provide direct reporting/tracker resources and organizing community advocacy and community relief and support resources in the wake of anti-Asian violence since the start of the pandemic.
South Asian Council for Social Services (SACSS)	Underserved South Asian and the broader immigrant community in NYC	Collaborating with NYC Health+Hospitals city hospital system to provide free, walk-in COVID-19 PCR testing and vaccination using mobile units (vaccine vans) and bilingual SACSS staff on-site in neighborhoods in Flushing, Queens, averaging ∼169 vaccinations a month between February and May 2021.^47^ Bilingual staff offering regular in-person and virtual information and resource provision, to ensure community members become familiar with health and economic resources available to them (NYC Care), build health and technological literacy, and deliver critical senior support and wellness services (averaging 7 support groups, 187 wellness calls, and 79 counseling sessions per month between February and May 2021) to low English-proficient community members. Provides emergency food relief since start of the pandemic, building on existing culturally relevant food pantry and social service programs to addresses food shortage and hunger, feeding 4800–5000 individuals/week. Offering free weekly grocery delivery of pantry staples to over 14,500 low-income individuals and cooked hot, South Asian meals for 450+ immigrant and low-income community members since start of pandemic. In 2020, distributed over 200,000 masks, hand sanitizer, and other PPE to individuals and families. Patient navigators and CBO-staff provide in-language phone-based health navigation and benefit assistance such as unemployment benefits, and making vaccination appointments.
UNITED SIKHS	Sikh community in NYC metropolitan area	Participating in NYC's Test & Trace Corps to bring free walk-in COVID-19 testing at local community sites across NYC. Between October 2020 to August 2021, hosted ∼71 in-person community testing events.^48^ Mobilized large-scale cooking resources at local gurdwaras across Queens, NY, to provide food aid outside their places of worship to community members.

ACA, Affordable Care Act; CBO, community-based organization; CDC, Centers for Disease Control and Prevention; CEAL, Community Engagement Alliance; CHC, community health center; CHW, community health worker; COVID-19, coronavirus disease 2019; FQHC, federally qualified health center; HHS, United States Department of Health and Human Services; KAMPANY, Korean American Physicians Association of NY; LEP, limited English proficiency; NH/PI, Native Hawaiian and Pacific Islander; NYC, New York City; NYU CSAAH, New York University Center for the Study of Asian American Health; PCR, polymerase chain reaction; PI-CoPCE, Pacific Islander Center of Primary Care Excellence; PPE, personal protective equipment; PSA, public service announcement; RNs; registered Nurses; SNAP, Supplemental Nutrition Assistance Program; UT, Utah.

The deliberate inclusion of community advisory boards such as the NAC ensures the effectiveness of evidence-based interventions in public health emergencies and the increased uptake of COVID-19 testing and vaccination among underresourced and historically marginalized communities who may be distrustful of government and health institutions,^23^ and vulnerable to COVID-19 misinformation.^24^

Engaging in community-driven research and evaluation may also help to build CBOs' organizational capacity and strengthen their overall programming to improve community health and well-being.^25^ CBOs are key stakeholders in the public health response and should be considered as such, particularly as they have filled the gaps in the fractured health care and polarized social service systems for already structurally vulnerable communities.

The next sections provide two case studies to make plain select CBO efforts to support community health throughout the COVID-19 pandemic. Although we focus on the role of Asian American serving organizations, we acknowledge the tireless efforts of NH/PI-serving organizations (e.g., National Tongan American Society [NTAS] and Papa Ola Lōkahi [POL]) that have equally driven multilevel, collaborative, community-centered efforts to bolster and strengthen the NH/PI communities in the face of the pandemic.

### Case study 1: South Asian Council for Social Services

Since 2000, the South Asian Council for Social Services (SACSS) has provided social services, senior programs, food pantry, economic benefits and insurance enrollment support, and civic engagement activities to community members in Queens, New York (NY), serving an estimated 50,000 clients each year. SACSs' overall mission aims to empower underserved South Asians and other immigrant community members (e.g., Chinese, Korean, African American, and Latinx immigrants) through social services to integrate into the civic and economic fabric of New York City (NYC). All community programs are free and provided by culturally competent staff members who speak 12 different South Asian languages, Mandarin, Cantonese, Hakka, Spanish, and Creole.

The COVID-19 pandemic devastated the Bangladeshi and South Asian immigrant population in NYC,^26^ many of whom are self-employed or are service workers (e.g. food vendors, cab drivers, and essential workers). A lack of translated social distancing and infection prevention guidance or coordinated state or city outreach to the Bangladeshi community during the early days of the pandemic meant that many were unaware and unprepared for the severity of the virus, experiencing immunocompromised working conditions, job layoffs and lack of work, exacerbating food insecurity, and language barriers to accessing available city resources for support and heightening vulnerability.^26^

SACSS' already popular weekly food pantry, the first and only dedicated South Asian food pantry in NYC, became an essential lifeline for many community members who lost family and/or jobs due to the pandemic.

In spring 2020, SACSS provided deliveries of prepared meals and packed grocery bags to community members in place of regular food pantry distribution days; they have since transitioned back to in-person community food pick-up in adherence to city and state COVID-19 safety guidelines. Grocery delivery continues as SACSS continues to receive referrals from City agencies, hospitals and CBOs. In direct response to food shortages and hunger in the community, SACSS fed an estimated 5000 individuals per week in 2020 with nutritious South Asian food staples while also supporting the South Asian and greater NYC community's emotional and social well-being.^27,28^

SACSS also began providing live virtual sessions to offer space for social connection, including live-demo “cooking shows” featuring culturally relevant food pantry items, and a well-attended mental health panel session for community members to share strategies for coping with pandemic-triggered grief, anxiety, despair, and trauma.

SACSS staff include certified navigators, counselors, social workers, and community health advocates, who quickly transitioned their regular services to virtual or phone channels in response to the NYC-wide lockdown in March 2020, and continued providing culturally and linguistically appropriate senior support, counseling services, and enrollment assistance with health insurance, rental, unemployment, and economic benefits. SACSS served more than 30,000 community members in 2020, who were struggling with essential needs during the COVID-19 pandemic, such as feeding their families, paying bills, and staying healthy.

In response to shifting city and state COVID-19 guidelines, SACSS extended its translation and interpretation support to include COVID-19 protective measures and increased on-the-ground, targeted outreach efforts to promote testing and vaccination. SACSS continues to collaborate with local community organizations and clinical providers to host educational events and virtual workshops and forums to provide community space for support and to facilitate community discussions related to COVID-19 misinformation, anti-Asian sentiment, mental health needs, and COVID-19 vaccination.

Since summer 2020, SACSS has provided mobile testing and vaccination units in collaboration with NYC's Test & Trace Corps, a partnership between NYC Health+Hospitals, the largest public hospital system in the United States, and the NYC Department of Health and Mental Hygiene (DOHMH), to bring testing and vaccination services to where community members live and work. SACSS staff joined local partner organizations and health department staff to provide critical, bilingual COVID-19 services to the community to increase COVID-19 testing, personal protective equipment (PPE) distribution, and vaccine access to community members across Queens, NY.^29^

SACSS' participation with the Test & Trace Corps initiative is a natural extension of its programmatic efforts and is built on partnerships with local community groups and city leaders to disseminate resources and advocate for greater social, economic, and health-related services for their community.

This partnership and the SACSS ongoing health resource and community support efforts, both in-person and via community-preferred social media channels, highlight how hyperlocal CBOs were compelled to quickly fill critical COVID-19- and health-related information and service gaps during, as well as before, the pandemic. In addition to COVID-19-related efforts, SACSS has sustained and even expanded its regular health, social, and senior programming and services to ensure linguistic and equitable health access for all New Yorkers, opening a newly developed, two-story community center in August 2021 to provide expanded health, social, and senior services to the primarily immigrant community and clientele in NYC.^30^

### Case study 2: Korean Community Services of Metropolitan New York, Inc. (KCS)

The Korean Community Services of Metropolitan New York, Inc. (KCS) has regularly provided culturally competent social services, family activity programs, and economic benefits and insurance enrollment support since its founding in 1973, in response to demand for these direct services and educational programs. Their sustained organizational growth and continued advocacy efforts spurred KCS to open the first NY state-licensed outpatient mental health clinic operated by a Korean nonprofit organization in 2015.

At the start of the pandemic in March 2020, KCS transitioned its in-person programs to virtual or phone platforms, including pivots from weekly on-site hot meals to meal delivery services, providing culturally competent hot meals and fresh produce directly to community members. Bilingual staff provided mental health support via socially distanced, in-person check-in visits with socially and linguistically vulnerable Korean senior community members, to reduce the effects of social isolation in the wake of the COVID-19 lockdown.

Through these continued community contact points, KCS staff were able to develop and disseminate tailored, virtual COVID-19 educational trainings created in partnership with the Korean American Physicians Association of NY (KAMPANY) and CSAAH to respond to community questions and concerns on topics related to general COVID-19-related information, COVID-19 and HBV, and COVID-19 and diabetes.

Through the spring and fall of 2020, KCS clinical staff and CHWs provided free COVID-19 testing events at KCS community sites, successfully preregistering and testing more than 1000 individuals across four events in May 2020 alone. KCS outreach events also focused on distributing PPE, providing up-to-date, prevention-related information on testing and infection in preferred languages and from trusted community members, and providing critical social supports.

In summer 2020, KCS initiated a partnership with NYC's Test & Trace Corps to launch polymerase chain reaction testing and organize neighborhood mobile testing events. Mobile vans were staffed by bilingual teams to engage the community and increase COVID-19 testing and vaccination access to historically underresourced and immigrant Asian American communities. In March 2021, NYC Mayor Bill de Blasio named KCS's primary community center site one of only several permanent community-based vaccination sites, and the only Asian American-serving CBO with this designation.^31^

KCS has successfully increased COVID-19 vaccine uptake with the local senior and immigrant populations, offering more than 160 appointments daily, 7 days a week with trusted providers, and in patient-preferred languages.^32^

In July 2021, KCS received support from the Vaccine Access Fund through the Local Initiatives Support Corporation (LISC) NY, to provide free transportation (Uber rides) to testing and vaccination sites to community members in neighborhoods with limited testing locations or transportation-related difficulties. Through the National Institutes of Health (NIH)-funded Community Engagement Alliance (CEAL) Against COVID-19 Disparities initiative, the KCS team has also partnered with trusted local faith-based organizations to strengthen community testing and provide critical COVID-19-related information and vaccine services at local places of congregation (e.g., faith-based organizations and small businesses), focusing on culturally tailoring COVID-19-related services to establish comfort and trust.

The NIH CEAL effort relies on community-engaged partnerships between local community organizations, messengers, and researchers working closely to coordinate COVID-19-related educational outreach, to address misinformation and mistrust, and to encourage COVID-19 testing, vaccine uptake, and clinical trial and research engagement in communities hit hardest by COVID-19.^33^

The combination of culturally tailored health care delivery and meeting community members where they are, in-service provisions, and community engagement and outreach highlights the important role of CBOs in advancing local, state, and national outreach to historically underserved and marginalized communities. “If people feel comfortable, they will bring their family and friends.”^34^

### Critical role in the prevention and maintenance of chronic conditions

CBOs' capacity to support the prevention and management of comorbidities has been well demonstrated^35^ and is relevant given the increased risk and severity for worse COVID-19 outcomes among older adults and individuals with chronic conditions.^36^ CBOs are well-positioned to launch effective, localized, tailored, and targeted health promotion approaches that complement population-level strategies. Evidence-based programs conducted by CBOs and faith-based organizations have increased patient engagement (e.g., health education and counseling, recruitment and outreach) in community-based settings.^35,37^ However, CBOs require adequate federal and local funding and technical assistance to sustain their programs.

Recent evidence suggests that Asian American and NH/PI CBOs' COVID-19-related needs have not been very well served. In 2020, 73% of Asian American and NH/PI community-serving organizations reported needing dedicated funding to support lost revenue, avoid shutdowns of existing programs, and avoid staff layoffs. As of summer 2021, only 39% of Asian American and NH/PI CBOs noted having received such needed funding.^38^ Community-driven advocacy for any funding underscores the importance and need of community-based programmatic work while capturing how CBO work is not being adequately or equitably supported by federal and state decision makers.

Prior evidence has established the unequal impact of chronic disease (e.g., diabetes and prediabetes, obesity, and hypertension) in Asian subpopulations.^39,40^ For example, there are striking diabetes disparities for Asian Americans in aggregate and in particular for South Asian Americans, who have a higher diabetes prevalence compared with non-Hispanic whites, other Asian subgroups, and other racial groups.^41^ Yet, despite the high prevalence rates, few culturally and linguistically adapted diabetes management interventions exist for this population.^41^ Together with lacking disaggregated Asian American data, rising trends and exacerbated prevalence of chronic conditions, such as obesity, among some Asian subgroups have been hidden.^40,42^

These challenges underscore the salience and value of collaborative efforts with CBOs to improve the availability of plain-language health information and resources that are adapted for Asian American populations, but also provide culturally congruent, common-sense interpretations of important health information.

The public health response requires greater investment in emergency preparedness and services that improve community-clinical linkages and navigation of complex health systems, particularly for CBOs that provide culturally and linguistically tailored programming to support COVID-19-related needs, as well as social and health challenges, and social determinants of health to Asian American and other racial/ethnic minority communities, without sustained or dedicated funding.^38^

## Reinforcing the Role of CBOs in the Public Health Response

Ensuring equitable allocation of the COVID-19 vaccine, boosters, and resources to historically underserved populations is critical for reducing mortality, particularly with the emergence of new COVID-19 variants. Vaccine “acceptance” should not be conflated with vaccine access or intention of receiving the vaccine, given the structural barriers to COVID-19 services for large segments of the population lacking sufficient language support.

As vaccine rollout continues, reinforcing the role of CBOs in public health communication and dissemination may improve vaccine uptake response in underresourced communities, particularly among low-income and low-LEP settings. Recent examples includes NYC's Council allocation of $4 million citywide to the AAPI Community Support Initiative that will directly support Asian American- and NH/PI-led and -serving organizations that provide critical services such as mental health support, racial literacy, and youth programs.^43^

The CBO capacity to outreach into local neighborhood communities may also bolster contact tracing efforts, serve as a cost-efficient, community-based workforce to disseminate culturally and linguistically tailored information on COVID-19, and concurrently offer direct connections to public and social services. Lastly, more emphasis should be placed on improving emergency reserves of cash for CBOs to aid in future emergency situations.

A multipronged plan is needed to confront the continued surges of COVID-19 and pandemic landscape. Health and governmental leaders must expand efforts to connect with and establish trust with CBOs. The pandemic has reminded us that continued assessment and quality improvement of health and social systems are essential to prepare for unprecedented disruptions and provide quality care for all populations. CBOs' unique ability to provide a breadth of services at multiple access points effectively facilitates better care coordination, ultimately benefiting all populations during the pandemic and public health emergencies.

## Abbreviations Used

AAHC: Asian American Health Coalition
ACA: Affordable Care Act
APCA: Asian Pacific Community in Action
APIAHF: Asian and Pacific Islander American Health Forum
CAMS: Chinese American Medical Society
CBOs: community-based organizations
CDC: Centers for Disease Control and Prevention
CEAL: Community Engagement Alliance
CHC: community health center
CHW: community health worker
COPO: Council of Peoples Organization
COVID-19: coronavirus disease 2019
CPACS: Center for Pan Asian Community Services
CSAAH: Center for the Study of Asian American Health
DOHMH: Department of Health and Mental Hygiene
FQHC: federally qualified health centers
HHS: United States Department of Health and Human Services
KAMPANY: Korean American Physicians Association of NY
LEP: limited English proficiency
LISC: Local Initiatives Support Corporation
NAC: National Advisory Committee
NHLBI: National Heart, Lung, and Blood Institute
NIH: National Institutes of Health
NIMHD: National Institute on Minority Health and Health Disparities
NTAS: National Tongan American Society
NY: New York
NYC: New York City
OCAPICA: Orange County Asian Pacific Islander Community Alliance
PCR: polymerase chain reaction
PI-CoPCE: Pacific Islander Center of Primary Care Excellence
POL: Papa Ola Lokahi
PPE: personal protective equipment
PSA: Public Service Annoucement
RCMAR: Resource Centers for Minority Aging Research
RNs: registered nurses
SACSS: South Asian Council for Social Services
SNAP: Supplemental Nutrition Assistance Program
UT: Utah
